# Dentate gyrus network dysfunctions precede the symptomatic phase in a genetic mouse model of seizures

**DOI:** 10.3389/fncel.2013.00138

**Published:** 2013-08-30

**Authors:** Oana Toader, Nicola Forte, Marta Orlando, Enrico Ferrea, Andrea Raimondi, Pietro Baldelli, Fabio Benfenati, Lucian Medrihan

**Affiliations:** ^1^Department of Neuroscience and Brain Technologies, Fondazione Istituto Italiano di TecnologiaGenoa, Italy; ^2^International Max-Planck Research School for NeurosciencesGöttingen, Germany; ^3^Department of Experimental Medicine, University of GenoaGenoa, Italy

**Keywords:** epilepsy, synapsins, dentate gyrus, mossy cells, excitatory inhibitory balance

## Abstract

Neuronal circuit disturbances that lead to hyperexcitability in the cortico-hippocampal network are one of the landmarks of temporal lobe epilepsy. The dentate gyrus (DG) network plays an important role in regulating the excitability of the entire hippocampus by filtering and integrating information received via the perforant path. Here, we investigated possible epileptogenic abnormalities in the function of the DG neuronal network in the Synapsin II (Syn II) knockout mouse (Syn II^−/−^), a genetic mouse model of epilepsy. Syn II is a presynaptic protein whose deletion in mice reproducibly leads to generalized seizures starting at the age of 2 months. We made use of a high-resolution microelectrode array (4096 electrodes) and patch-clamp recordings, and found that in acute hippocampal slices of young pre-symptomatic (3–6 week-old) Syn II^−/−^ mice excitatory synaptic output of the mossy fibers is reduced. Moreover, we showed that the main excitatory neurons present in the polymorphic layer of the DG, hilar mossy cells, display a reduced excitability. We also provide evidence of a predominantly inhibitory regulatory output from mossy cells to granule cells, through feed-forward inhibition, and show that the excitatory-inhibitory ratio is increased in both pre-symptomatic and symptomatic Syn II^−/−^ mice. These results support the key role of the hilar mossy neurons in maintaining the normal excitability of the hippocampal network and show that the late epileptic phenotype of the Syn II^−/−^ mice is preceded by neuronal circuitry dysfunctions. Our data provide new insights into the mechanisms of epileptogenesis in the Syn II^−/−^ mice and open the possibility for early diagnosis and therapeutic interventions.

## Introduction

Epilepsy is a debilitating nervous system disorder mainly characterized by abnormal synchronization of neuronal activity. Current treatments are often unsatisfactory and have undesirable side effects and a number of patients present intractable seizures, for which surgical treatment is the only option (Wilson et al., 1977). This clearly indicates that mechanistically epileptic seizures are far from being understood.

The hippocampal formation is part of the limbic system and represents an important integration site, receiving inputs from most brain areas mainly via the perforant path. Intractable medial temporal lobe epilepsies (MTLE) are often successfully treated by excision of the hippocampus and nearby regions of the brain (Schwartzkroin, 1994). Therefore, this brain area appears to be highly seizure-prone and provides a convenient model to study epileptiform events *in vitro*. The major subdivisions of the hippocampus are the dentate gyrus (DG) and the *cornu ammonis* (CA), further divided in the CA3-CA1 regions. The DG has stemmed a lot of interest due to its densely packed, hyperpolarized granule cells thought to have a gate role, filtering synaptic input mainly received from the perforant path (Amaral et al., 2007). Granule cells are locally regulated by excitatory hilar mossy cells and by several types of inhibitory interneurons (Amaral et al., 2007; Hsu, 2007). There are several theories that propose mossy cells to be key players in hippocampal seizure-like events (Scharfman and Myers, 2012). These are based on several mossy cell characteristics: (i) they are highly excitable and receive input from thousands of granule cells; (ii) they are very sensitive to excitotoxicity; (iii) their axonal projections extend up to millimeters along the septo-temporal axis of the hippocampus, making them suitable candidates for seizure spread; (iv) temporal-lobe epilepsy is often associated with hilar cell loss in patients and in some laboratory models (Santhakumar et al., 2000; Ratzliff et al., 2002; Jinde et al., 2012, 2013; Scharfman and Myers, 2012).

There are several animal models widely used in the study of seizures and epilepsy, both *in vitro* and *in vivo*. Acute pharmacological models are obtained mostly by increasing excitation or lowering inhibition (blocking K^+^-channels with 4-aminopyridine, using a low Mg^2+^ concentrations in the recording solution to relieve the NMDA receptor Mg^2+^ block, treatment with bicuculline or penicillin to block GABA_A_ receptors) (Gutnick et al., 1982; Voskuyl and Albus, 1985; Witte, 1994; Westerhoff et al., 1995). Chronic models are obtained with kainic acid injection, leading to loss of CA3 neurons in the hippocampus (Nadler, 1987), or with pilocarpine injection, causing excessive stimulation of M1 muscarinic receptors in the hippocampus and subsequent neuronal loss (Turski et al., 1983). Another murine epilepsy model, the so-called “kindling” model is based on the finding that repeated seizures can lead to development of epilepsy. Experimental kindling can be triggered, either electrically or chemically, by inducing repeated, short, focal seizures that eventually lead to the appearance of more severe, chronic seizures (Goldberg and Coulter, 2013). The molecular mechanism of kindling is unclear, but there are data supporting the role of BDNF upregulation in the progression of kindling (Garriga-Canut et al., 2006). In all these models of chemically or electrically induced epilepsy, as well as in other models (e.g., stroke or traumatic brain injury), the process of epileptogenesis starts within minutes to months and requires neuroanatomical changes, neural network alterations, activation of inflammatory cascades, post-translational modifications of existing proteins, activation of immediate early genes and several transcriptional changes (Rakhade and Jensen, 2009; Hunt et al., 2013). One comprehensive study evaluated the change in RNA expression across all annotated rat genes after pilocarpine treatment (Okamoto et al., 2010). Overall, there were about 1400 genes that changed their expression throughout the progression of epileptogenesis, with a group of 128 genes that were found to be consistently overexpressed at all stages. The proteins encoded by these genes were involved in the immune response, cell motility, apoptosis, and intracellular signaling cascades (including the mTOR signaling pathways) (Okamoto et al., 2010; Goldberg and Coulter, 2013). This study suggests that epileptogenesis is a highly complex process, involving very diverse groups of genes.

Epilepsy is an inheritable condition and mutations in several genes are associated with seizure disorders in human patients (Poduri and Lowenstein, 2011). Genetic manipulations of these target genes in mice have provided mouse models of epilepsy that are of invaluable help for the understanding of the neuropatophysiological changes in epileptogenesis (Mantegazza et al., 2010). One of the family of genes mutated in several epileptic patients, synapsins (Syns), are a family of abundant neuronal phosphoproteins playing important roles in synaptogenesis, synaptic transmission and plasticity. In mammals, they are encoded by three related genes, *SYN1-3* (Kao et al., 1999), translated into ten protein isoforms through alternative splicing (Syn Ia/Ib, Syn IIa/IIb, and IIIa/IIIf) (Cesca et al., 2010; Fassio et al., 2011). The best described function of Syns is the control of neurotransmitter release by clustering synaptic vesicles (SVs) and reversibly tethering them to the actin cytoskeleton, thus maintaining the integrity of the recycling pool (Benfenati et al., 1992). Upon activity-dependent phosphorylation, Syns detach from SVs and render them free to fuse with the plasma membrane (Giovedi et al., 2004; Menegon et al., 2006; Messa et al., 2010). This is an oversimplified view, since experiments have shown that the regulation of neurotransmitter release by Syns is much more subtle and finely tuned (Cesca et al., 2010; Fassio et al., 2011). In both excitatory and inhibitory synapses Syns appear to control downstream events of SV exocytosis, such as docking and fusion and, implicitly, the size of the readily releasable pool (RRP) (Baldelli et al., 2007; Gitler et al., 2008; Valente et al., 2012; Medrihan et al., 2013).

All mutant mice lacking one or more Syn isoforms are prone to epileptic seizures, except for Syn III^−/−^ mice (Cesca et al., 2010) with Syn II^−/−^ mice showing the strongest epileptic phenotype (Etholm et al., 2012). Genetic mapping analysis identified SYN2 among a restricted number of genes significantly contributing to epilepsy predisposition (Cavalleri et al., 2007; Lakhan et al., 2010). Synapsin II appears to have a specific role in preventing synaptic depression and maintaining the SV recycling pool at central excitatory synapses and at the neuromuscular junction (Rosahl et al., 1995; Coleman et al., 2008). Moreover, in DG inhibitory synapses, Syn II controls the dynamics of neurotransmitter release leading to an increase in the RRP responsible for the synchronous release in the detriment of the asynchronous GABA release (Medrihan et al., 2013). Syn II, but also Syn I knockout mice (Syn I^−/−^ and Syn II^−/−^) present mild emotional memory deterioration as they age (Greco et al., 2013), and are highly seizure prone. After 2–3 months of age, both Syn II^−/−^ mice and Syn I^−/−^ mice become epileptic and exhibit partial, secondarily generalized epileptic seizures triggered by novelty stimuli such as handling, loud noise or mating (Etholm et al., 2012). One of the most important questions regarding the Syn II^−/−^ mouse epileptic model concerns the absence of seizures before the age of 2 months. Our aim in this study was to investigate the neuronal function in the pre-symptomatic phase of this mouse model. Given the large body of experimental evidence pointing toward the importance of the DG in epileptogenesis, here we have searched for pathological impairments of its circuitry in pre-symptomatic Syn II^−/−^ mouse brain slices, using a combination of electrophysiological and morphological techniques.

## Materials and methods

### Animals

Experiments were performed on 3–6 weeks (pre-symptomatic animals) or 4–6 months-old (after seizure onset) homozygous Syn II knockout (Syn II^−/−^) mice generated by homologous recombination and age-matched C57BL/6J wild-type (WT) animals. All experiments were carried out in accordance with the guidelines established by the European Community Council (Directive 2010/63/EU of September 22nd, 2010) and were approved by the Italian Ministry of Health. Adequate measures were always taken to minimize animal pain or discomfort.

### Preparation of slices

Mice were anesthetized with isofluran by inhalation, decapitated and the brain dissected out in ice cold cutting solution containing (mM): 87 NaCl, 25 NaHCO_3_, 2.5 KCl, 1.25 NaH_2_PO_4_, 0.5 CaCl_2_, 7 MgCl_2_, 25 glucose, and 75 sucrose saturated with 95% O_2_ and 5% CO_2_. Horizontal, 400 μm thick, cortico-hippocampal slices were cut using a Microm HM 650 V vibratome equipped with a Microm CU 65 cooling unit (*Thermo Fisher Scientific, Waltham MA, USA*). Slices were cut at 2°C in the bath solution. After cutting, slices were left to recover for 45–60 min at 35°C and for another hour at room temperature in artificial cerebrospinal fluid (aCSF) containing in mM: 125 NaCl, 25 NaHCO_3_, 25 glucose, 2.5 KCl, 1.25 NaH_2_PO_4_, 2 CaCl_2_, and 1 MgCl_2_ (bubbled with 95% O_2_–5% CO_2_). The same solution was used for the perfusion of slices during recordings.

### Patch-clamp recordings

Whole-cell recordings were performed with a Multiclamp 700B/Digidata1440A system (*Molecular Devices, Sunnyvale, CA*) using an upright BX51WI microscope (*Olympus, Tokyo, Japan*). For all experiments we used a high-K gluconate intracellular solution containing (in mM): 126 K gluconate, 4 NaCl, 1 MgSO_4_, 0.02 CaCl_2_, 0.1 BAPTA, 15 Glucose, 5 HEPES, 3 ATP, and 0.1 GTP. The pH was adjusted to 7.3 with KOH and osmolarity was adjusted to 290 mosmol/l using sucrose. Patch-pipette resistance was 3–6 MΩ when filled with intracellular solution. Somatic access resistance (R_a_) was continuously monitored, and cells with unstable R_a_ (20% changes) or with values larger than 15 MΩ were excluded from the analysis.

Mossy cells were identified by their shape, which has a triangular appearance in an infrared differential interference contrast image, size (mossy cells are clearly larger that surrounding interneurons) and location (deep hilus). Mossy cells were also filled with AlexaFluor 568 (40 μM in the pipette solution) to make the specific complex spines (named thorny excrescences) on the proximal dendrites visible in epifluorescence imaging (see Figure 4C). For cells that could not be clearly visualized, electrophysiological features, such as firing frequency adaptation during positive current injection, reduced afterhyperpolarization, broad action potentials, and high frequency/large amplitude spontaneous excitatory postsynaptic currents (sEPSPs) in the presence of bicuculline/CGP 55845, were used to identify them. Intrinsic cell properties were calculated from recordings performed in the presence of 50 μM D-APV, 10 μM CNQX, 5 μM CGP 55845, and 30 μM bicuculline. For the recording of miniature excitatory postsynaptic currents (mEPSCs), aCSF cointaining 5 μM CGP 55845, 30 μM bicuculline, and 0.3 μM TTX was used. All experiments were performed at a holding potential (*V*_h_) of -70 mV in the presence of 30 μM bicuculine and 5 μM CGP 55845 (all from *Tocris Bioscience, Ellisville, MO*).

Granule neurons were selected based on their oval shape and their middle position in the granule layer. Since dentate granule neurons can be in different stages of maturation we recorded only mature neurons in which *R_m_* < 300 MΩ (Liu et al., 1998).

In experiments where extracellular stimulation was performed, a monopolar stimulation electrode (a glass pipette filled with aCSF) was placed in the hilus, close to the granule cell layer and connected to an external stimulator (*A-M Systems, Sequim WA*).

### Patch-clamp data analysis

All data were acquired with Clampex and analyzed offline with Clampfit 10.2 (*Molecular Devices, Sunnyvale CA, USA*), MiniAnalysis (*Synaptosoft, Decatur GA, USA*), Excel and GraphPad.

In the current-clamp mode, 30 current steps lasting 1 s, starting from −100 pA in 10 pA increments, were applied to both granule and mossy cells. To precisely determine the resting membrane potential (*V_m_*) value, this was continuously recorded for 1 min, plotted in a histogram, and fitted with a Gaussian curve. *V_m_* was taken as the mean of the distribution. To determine the *start* (*V_s_*) *of an action potential (AP)*, phase plane plots were constructed and *V_s_* was considered the voltage point where dV/dt exceeded 10 mV/ms. The AP amplitude was measured as the difference between the maximum voltage reached during the overshoot minus *V_s_*. The afterhyperpolarization (AHP) value was considered as the point where *dV/dt* was equal to zero, after the repolarization phase. The *rheobase* was estimated as the smallest current step that elicited an AP. For calculating the *input resistance* (*R*_in_), the steady state voltage values from the first step above and below zero current (−10 and +10 pA), plus the zero current trace were plotted against the injected current and fitted with a linear regression. *R*_in_ was taken as the slope of the fitting line. The coefficient of determination R^2^ was always higher than 0.95.

For the analysis of mEPSCs, 30 s of each recording were imported into MiniAnalysis (*Synaptosoft Inc., New Jersey*). Events' peaks were identified manually because of the presence of multipeak events due to the high frequency of spontaneous events. Subsequently, the software automatically calculated amplitude, rise, and decay. Minimum amplitude threshold was set to 8 pA. For the analysis of mEPSC kinetics, a set of individual 40–60 events was chosen in which the rise and the decay phase were very clear. The events were all aligned at the point of 50% rise and averaged for further calculations. The decay phase was best fitted with a monoexponential equation on the 90–10% decay phase. The cumulative distributions of amplitudes and frequencies were constructed by pooling all values, and analyzed with a Kolmogorov–Smirnov test for distributions.

### High-density active-pixel-sensor microelectrode array (MEA) recordings

The APS-MEA, extensively described elsewhere (Ferrea et al., 2012), consists of a microelectrode array chip and an amplification system designed to provide simultaneous extracellular recordings from 4096 electrodes at a sampling rate of 7.7 kHz. Each square pixel measures 21 × 21 μm, and the array is integrated with an electrode pitch (center-to-center) of 42 μm. Pixels are arranged in a 64 × 64 array configuration, yielding an active area of 7.22 mm^2^, with a pixel density of 567 pixels/mm^2^. The three on-chip amplification stages provide a global gain of 60 dB, with a 0.1–5 kHz band-pass filter. This bandwidth is suitable for recording both slow LFP signals and fast APs. The acquisition is controlled by the BrainWave software (*3Brain Gmbh, Switzerland*). For stimulation experiments of the DG, a monopolar stimulation electrode placed on the medial perforant path and connected to an external stimulator (*A-M Systems, Sequim WA*) was used. Clear evoked responses separated from the stimulation artifact were obtained using stimulation intensities between 100 and 500 μA (for 30 μs). Large-scale field recordings were acquired using BrainWave and analyzed offline with MatlabR2010a, Clampfit 10.2 and ImageJ. For the analysis of the peak amplitude, 3 representative channels (named “pixels”) from the molecular layer and 3 from the hilus were exported in Matlab (MathWorks, http://www.mathworks.it/) and then imported in Clampfit for analysis. The maximum amplitude of the evoked events on each channel was measured, averaged (10 events/experiment) and normalized to the control amplitude. For the analysis of the mean amplitude for the entire area, an image at the point of highest response for the respective area was imported in ImageJ and the mean intensity was calculated after normalization of the maximum intensity to the maximum voltage of the same response.

### Immunofluorescence

WT and Syn II^−/−^ mice were deeply anesthetized with 20% urethane (0.1 ml/10 gm) and perfused transcardially with 0.1 M phosphate buffer containing 4% paraformaldehyde (pH 7.4). Brains were subsequently postfixed overnight in paraformaldehyde solution, and then washed in PBS, infiltrated with a 30% sucrose PBS solution for cryoprotection and frozen in OCT. Ten μm thick horizontal sections were cut using a Leica CM3050 S Cryostat and collected on SuperFrost slides. Sections were incubated 1 h in blocking buffer (2% NGS, 1% BSA, 0.1% CFG, 0.1% Triton X-100, 0.05% Tween in PBS), then for 2 h in blocking buffer containing primary antibodies. After several washes in PBS, sections were incubated with blocking buffer containing fluorochrome-conjugated secondary antibodies (*Invitrogen*, 1:500), washed and mounted using Prolong Gold antifade reagent with DAPI staining (*Invitrogen*) for fluorescence microscopy observation on a Leica SP5 confocal laser scanning fluorescence microscope. The mean fluorescence intensity ratio between Syn I or Syn II and calretinin were calculated with the software ImageJ. Antibodies used were: Anti-Synapsin 1 (Mouse, 1:500, SYSY #106 001; Rabbit, 1:200 G-177), Anti-Synapsin 2 (Mouse, 1:200, clone 19.21), Anti-panSynapsins (Rabbit, 1:500 G143), Anti-Calretinin (Guinea Pig, 1:1000, SYSY# 214 104).

### Electron microscopy

Acute slices were fixed by immersion in 1.3% glutaraldehyde in 66 mM sodium cacodylate buffer. Subsequently, slices were post-fixed with 1% OsO4 in 1.5% K_4_Fe(CN)_6_ in 0.1 M sodium cacodylate, en bloc stained with 0.5% uranyl acetate, dehydrated through a series of graded ethanol solutions, washed in propylene oxide and flat embedded in Embed 812 between two Aclar sheets. After 48 h of polymerization at 60°C, a small region corresponding to the DG was excised, glued with cyanoacrylate glue on blocks of resin and cut with a Leica EM UC6 ultramicrotome. Ultrathin sections (thickness: 70–90 nm) were collected on Formvar carbon coated copper grids. Grids were observed in a JEOL JEM-1011 microscope operating at 100 kV using an ORIUS SC1000 CCD camera (*Gatan*). Total and docked SV densities were calculated using the software ImageJ.

### Statistical analysis

All data are expressed as means ± s.e.m. All statistics were performed with GraphPad Prism. For comparison between WT vs. Syn II^−/−^ experiments, two-tailed unpaired Student's *t*-test was used. For multiple comparisons, one or two-way analysis of variance (ANOVA) with Bonferroni *post-hoc* test or Kruskal-Wallis test followed by Dunn's *post-hoc* test was used, depending on the type of data. The level of significance was set at *p* < 0.05.

## Results

### Network impairments of DG in pre-symptomatic Syn II^−/−^ mice

**Figure 1:**
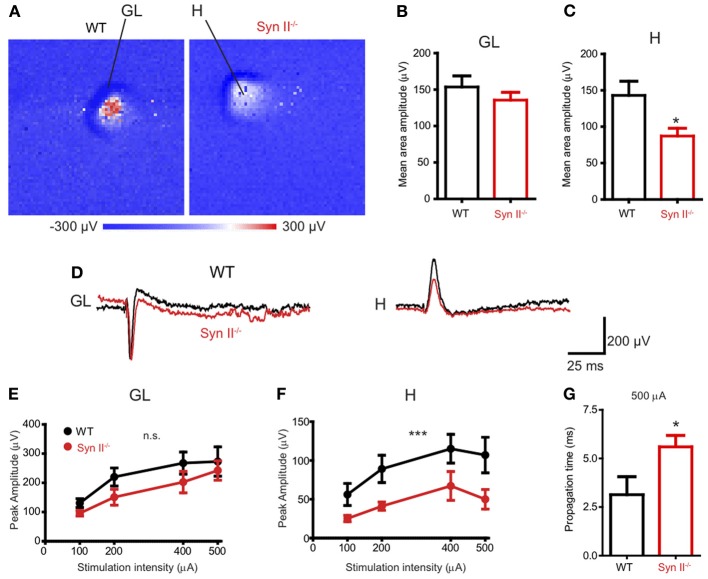
**APS-MEA extracellular field recordings show decreased activity in the hilus upon stimulation of the perforant path. (A)** Color-coded fPSP activity in the entire APS-MEA chip (each pixel represents one electrode) in WT and pre-symptomatic Syn II^−/−^ slices with arrows showing the activated areas (GL, granule layer; H, hilus) upon perforant path stimulation. **(B,C)** Mean (±s.e.m.) amplitude of the entire response area from WT and pre-symptomatic Syn II^−/−^ in the granule layer **(B)** and hilus **(C)**; ^*^p < 0.05, two-tailed unpaired Student's *t*-test. **(D)** Representative traces from one APS-MEA electrode from both genotypes located in the granule layer and hilus, respectively. **(E,F)** Stimulation-response curves representing the mean (±s.e.m.) peak amplitudes of three randomly selected electrodes from the granule layer **(E)** or hilus **(F)** at increasing stimulation intensities; ^***^p < 0.001, Two-Way ANOVA. **(G)** Mean (±s.e.m.) stimulus propagation time from GL to H at a stimulation intensity of 500 μA; ^*^p < 0.05, two-tailed unpaired Student's *t*-test.

Our first step was to investigate the response of different areas of the DG to the stimulation of the perforant path in horizontal brain slices containing the hippocampus and rhinal cortices from pre-symptomatic (3–6 weeks) Syn II^−/−^ mice. To this aim, we employed a high-resolution Active Pixel Sensor microelectrode array system (APS-MEA, 4096 electrodes: see Materials and Methods) (Ferrea et al., 2012). Field postsynaptic potentials (fPSPs), evoked by the stimulation of the perforant path with an extracellular electrode, were detected in the granule layer of the DG and they further propagated to the hilus (Figure 1A). The mean amplitude of the response, calculated over the entire activated region in the granule cell layer, was similar in WT and Syn II^−/−^ slices (153.7 ± 15.2 μV for WT vs. 135.6 ± 10.6 μV for Syn II^−/−^, *n* = 13 slices for each genotype; two-tailed unpaired Student's *t*-test, *p* = 0.342) (Figures 1B,D). On the other hand, the mean amplitude of the response in the hilar region was significantly reduced in Syn II^−/−^ slices (143.0 ± 19.4 μV for WT vs. 87.13 ± 10.7 μV for Syn II^−/−^, *n* = 12 slices for each genotype; two-tailed unpaired Student's *t*-test, *p* = 0.013) (Figures 1C,D). The stimulus amplitude was set to 400–500 μA based on previous input-output curves performed for 3 selected electrodes in each of the two regions. At all stimulation intensities, a reduced amplitude of the responses in the Syn II^−/−^ hilar region with respect to the WT was observed (Figures 1E,F). The recorded signal on several neighboring electrodes on the APS-MEA correlated with the fine anatomy of the DG and its polarity corresponded to current sinks in the dendritic-granule layer (negative) and to current sources in the hilus (positive) (Figure 1D). Moreover, the propagation time of the evoked fPSP, measured between the peaks of the response from one representative electrode in the granule and hilar regions, was significantly longer in Syn II^−/−^ than in WT slices (2.8 ± 0.78 ms for WT vs. 5.6 ± 0.59 ms for Syn II^−/−^, *n* = 6/5 slices; two-tailed unpaired Student's *t*-test, *p* = 0.032) (Figure 1G), suggesting the presence of functional impairments of the hilar region.

**Figure 2:**
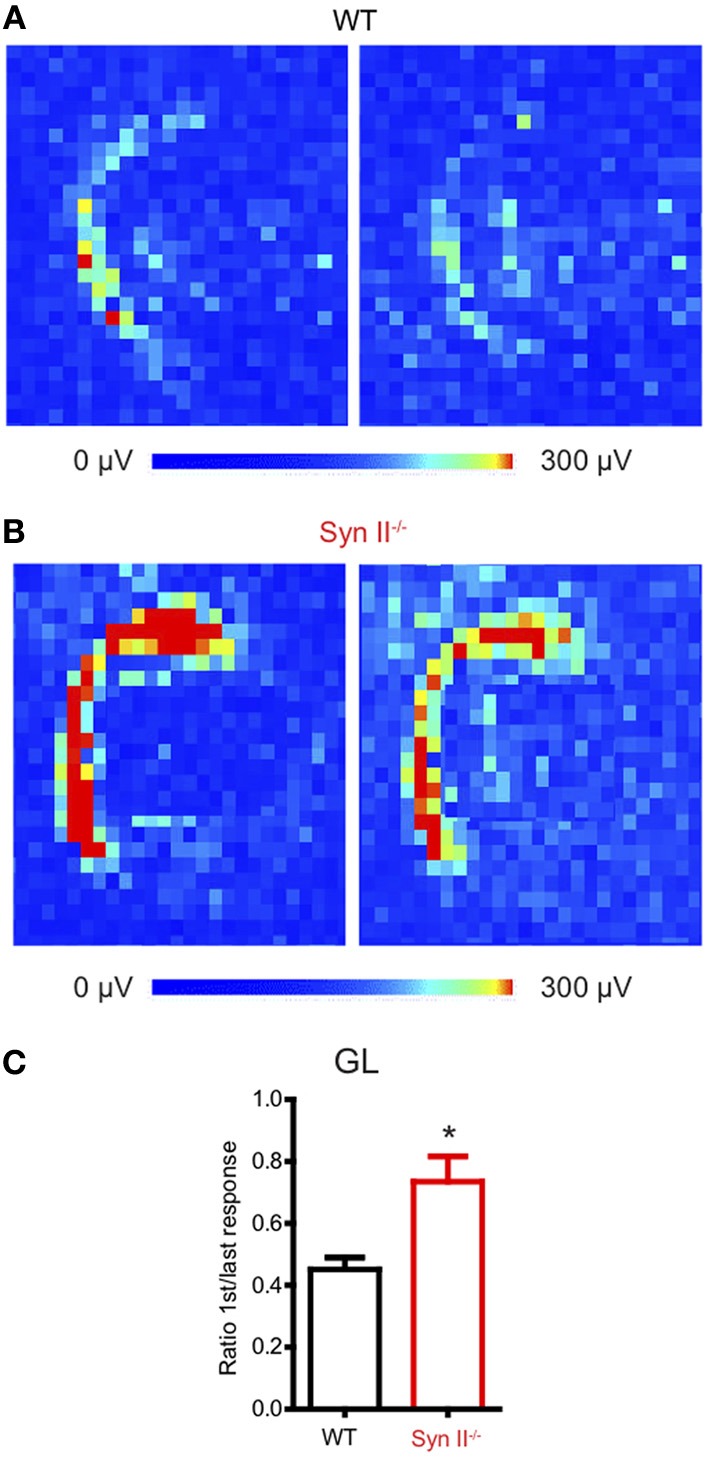
**Perforant path depression is reduced in slices from young Syn II^−/−^ mice. (A,B)** Color-coded fPSP activity on a selected area on the APS-MEA chip representing the response of the DG granule layer from WT **(A)** and pre-symptomatic Syn II^−/−^ **(B)** slices to the first (left) and the last (right) perforant path extracellular stimulation in a train of 5 s at 20 Hz. **(C)** Mean (±s.e.m.) ratio between the mean granule layer amplitude of the first and the last response in the train for both genotypes; ^*^p < 0.05, two-tailed unpaired Student's *t*-test.

The hilus of the DG contains hilar mossy cells and inhibitory interneurons, whose activity modulate the excitability of granule neurons (Scharfman and Myers, 2012), by forming a regulatory loop. We have previously shown that the plasticity of the young Syn II^−/−^ excitatory and inhibitory synapses upon train stimulation of the perforant path does not significantly differ from that of WT synapses (Medrihan et al., 2013). Since we observed a reduced signal in the hippocampal hilus, we reasoned that a sustained train of stimuli would reveal possible dysfunctions of the granule layer-hilus network function. Thus, we stimulated the perforant path with a train of 20 Hz for 5 s and measured the ratio between the first and the last evoked fPSP response of the train over the entire granule cell layer area (Figures 2A,B). To our surprise, the depression induced by a train of stimuli was significantly higher in WT than in Syn II^−/−^ slices (0.45 ± 0.03 for WT vs. 0.73 ± 0.08 for Syn II^−/−^, *n* = 4/5 slices; two-tailed unpaired Student's *t*-test, *p* = 0.033) (Figure 2C). Based on previous results showing that perforant path plasticity is unaltered in Syn II^−/−^, these results point toward a reduced feedback inhibition of the granule cell layer.

### Synapsin II is abundantly expressed in terminals of both granule and mossy neurons

**Figure 3:**
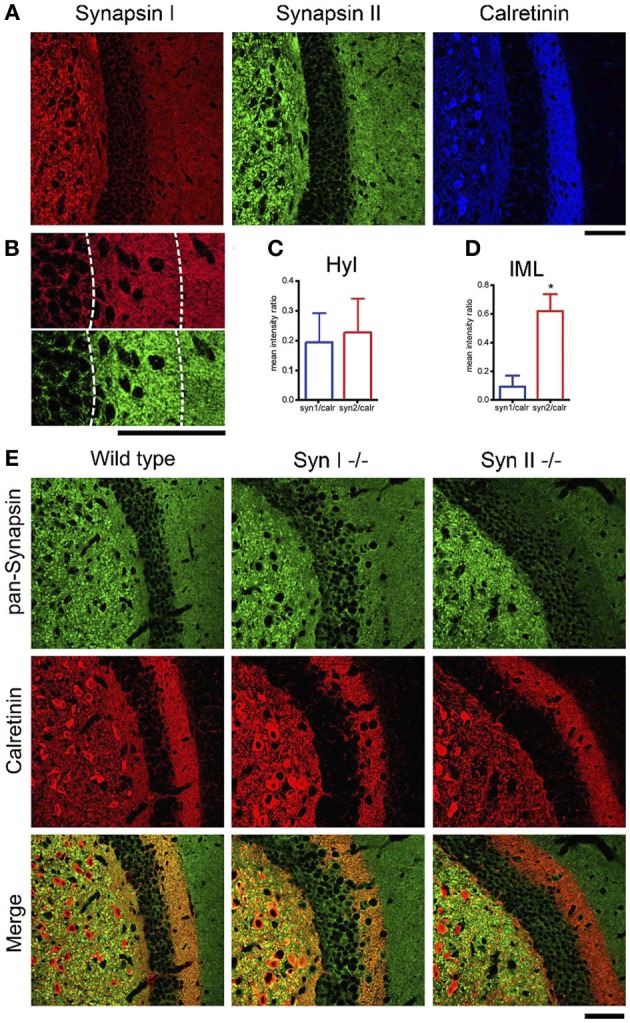
**SynII is highly colocalized with the mossy cell marker calretinin. (A)** Representative images of the DG region in brain slices from 6 weeks old WT mice immunostained for Syn I, Syn II, and calretinin (scale bar 50 μm); **(B)** Representative images of the inner molecular layer area (IML) of the DG immunostained for Syn I (upper panel) or Syn II (lower panel) (scale bar, 50 μm); **(C,D)** Mean (±s.e.m.) ratios between Syn I/calretinin and Syn II/calretinin signals in the hilus (Hyl, **C**) and inner molecular layer (IML, **D**) of the DG. ^*^p < 0.05, two-tailed unpaired Student's *t*-test. **(E)** Representative images of the DG region in brain slices from 6 weeks old WT (left column), Syn I^−/−^ (middle column) or Syn II^−/−^ (right column) mice immunostained for all synapsin isoforms (pan-Syn antibody) and calretinin. Right panels display the merge of the two signals (scale bar, 50 μm).

Next, we confirmed the presence of Syn II in the DG. Acute brain slices from 6 weeks old WT mice were immunolabeled for endogenous Syns I and II and for the mossy cell marker calretinin (Blasco-Ibanez and Freund, 1997) (Figure 3A). Since in some species calretinin is also a marker for GABAergic interneurons (Scharfman and Myers, 2012), we co-immunolabeled for GABA and noticed that calretinin immunoreactive hilar cells are negative for GABA (data not shown), suggesting that they are indeed hilar mossy cells. Both Syn I and Syn II puncta were abundantly observed in the hilar region of the DG, where mossy fiber terminals are localized. Remarkably, Syn II, but not Syn I, positive puncta were present in high density in the inner molecular layer, colocalizing with the hilar cell terminals. We therefore measured the mean intensity ratio between either Syn isoform and calretinin in the inner molecular layer of the DG (0.09 ± 0.007 for Syn I/calretinin and 0.61 ± 0.11 for Syn II/calretinin, *n* = 3 mice; two-tailed unpaired Student's *t*-test, *p* = 0.034) and in the hilar region (0.195 ± 0.0994 for Syn I/calretinin and 0.229 ± 0.1157 for Syn II/calretinin, *n* = 3 mice; two-tailed unpaired Student's *t*-test, *p* = 0.837) (Figures 3B–D). To further prove the specific presence of Syn II at mossy cell terminals, we stained brain slices from Syn I^−/−^ and Syn II^−/−^ mice respectively for calretinin (red) and all Syn isoforms (pan-synapsin antibody, green). While in the Syn I^−/−^ slices the pan-synapsin staining is visible in the IML, it was completely absent in the same region of the Syn II^−/−^ slices (Figure 3E, bottom panels).

### Reduced synaptic activity in mossy cells from pre-symptomatic Syn II^−/−^ mice

**Figure 4:**
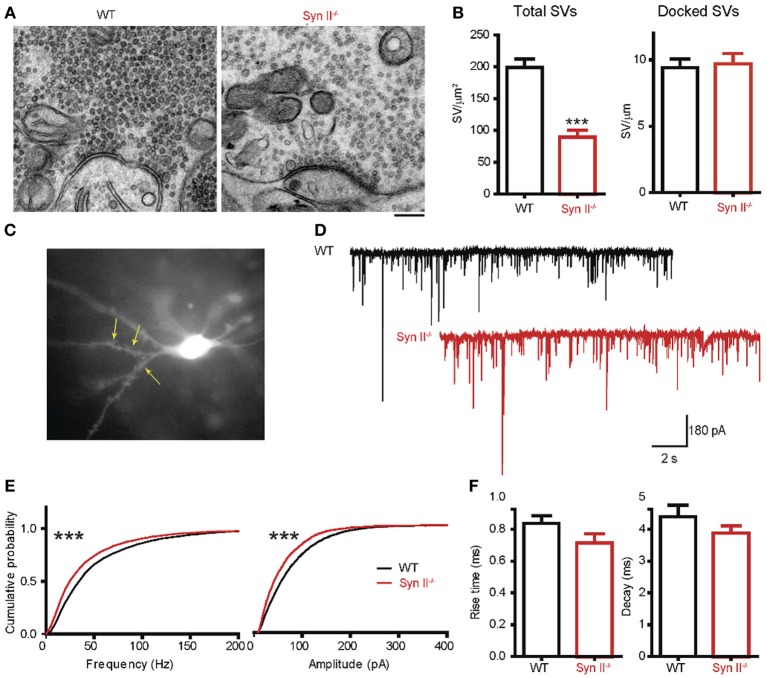
**A decreased number of mossy fiber synaptic vesicles is associated with reduced mEPSC frequency and amplitude in mossy cells from pre-symptomatic Syn II^−/−^ mice. (A)** Transmission electron microscopy images of mossy fiber terminals in the DG hilus of brain slices from WT (black bars) and pre-symptomatic Syn II^−/−^ (red bars) mice (scale bar 200 nm). **(B)** Mean (±s.e.m.) density of total SVs and number of docked SVs in presynaptic terminals of WT and Syn II^−/−^ neurons; ^***^p < 0.001, two-tailed unpaired Student's *t*-test. **(C)** Representative Syn II^−/−^ hilar mossy cell patch-clamped in an acute brain slice and filled with AlexaFluor568. Yellow arrows point toward thorny excrescences that are visible in the focal plane. **(D)** Representative mEPSC traces recorded in mossy cells from WT (black lines) and pre-symptomatic Syn II^−/−^ (red lines) mice in the presence of GABA receptor and Na^+^ channel blockers. **(E)** Cumulative distributions of the amplitudes and frequencies of mEPSCs in WT and Syn II^−/−^ neurons; ^***^p < 0.001, Kolmogorov–Smirnov test. **(F)** Mean (±s.e.m.) rise-time (10–90%) and mono-exponential τ of decay of mEPSCs from WT (black bars) and Syn II^−/−^ (red bars) neurons.

Since we noticed an impaired function of the hilar region of the DG (Figure 1) and Syn II is highly expressed at the mossy fiber terminals (Figure 3), we next investigated the synaptic input received by hilar mossy cells from granule neurons. First, we used electron microscopy to morphologically evaluate the number and spatial distribution of SVs within the mossy fiber terminals in the hilar region of the DG of pre-symptomatic Syn II^−/−^ and WT mice (Figure 4A). The total density of SVs in Syn II^−/−^ synapses was significantly lower than that of WT synapses (198.7 ± 13.7, *n* = 44 terminals/3 mice and 89.27 ± 10.8 SVs/μm^2^, *n* = 51 terminals/3 mice, for WT and Syn II^−/−^ respectively; two-tailed unpaired Student's *t*-test, *p* = 0.0003; Figures 4A,B). However, the number of docked SVs did not differ between genotypes (9.42 ± 0.6, *n* = 19 terminals/3 mice and 9.70 ± 0.7 SVs/μm, *n* = 19 terminals/3 mice, for WT and Syn II^−/−^ respectively; two-tailed unpaired Student's *t*-test, *p* = 0.782) (Figures 4A,B), as previously reported for Syn deletions at various synapses (Gitler et al., 2004; Medrihan et al., 2013).

To functionally analyze these synapses, we recorded mEPSCs from pre-symptomatic Syn II^−/−^ hilar mossy cells after blocking GABA receptors and Na^+^ channels with bicuculline (30 μM), CGP55845 (5 μM) and TTX (0.3 μM). To distinguish mossy cells from the surrounding hilar neurons, we filled the patch pipette with AlexaFluor568-containing intracellular solution. This enabled the detection of moss-resembling spines (called “thorny excrescences”) present on the proximal dendrites of these cells (Figure 4C). As reviewed before (Henze and Buzsaki, 2007; Scharfman and Myers, 2012), hilar mossy cells are highly excitable, receiving massive input from the mossy fibers, and their mEPSCs have an unusually large amplitude and frequency in comparison with other central synapses (Figure 4D). Both the amplitude and the frequency distributions of mEPSCs were significantly shifted toward smaller values in Syn II^−/−^ hilar mossy neurons (*n* = 11 neurons/7 mice for WT and 6 neurons/3 mice for Syn II^−/−^; Kolmogorov–Smirnov test, *p* < 0.001) (Figure 4E). Such a leftward shift of frequencies indicates the presence of a presynaptic involvement. Although the distribution of mEPSC amplitudes in Syn II^−/−^ was also left-shifted, it was not associated with a change in the kinetic parameters of the response (rise-time 10–90%: 0.83 ± 0.04 vs. 0.71 ± 0.05 ms, *p* = 0.168; decay τ: 4.39 ± 0.35 vs. 3.87 ± 0.23 ms, *p* = 0.374; two-tailed unpaired Student's *t*-test) (Figure 4F), suggesting an overall decreased excitatory presynaptic input on Syn II^−/−^ hilar mossy cells from young mice.

### Hilar mossy cells of pre-symptomatic Syn II^−/−^ mice display decreased excitability

**Figure 5:**
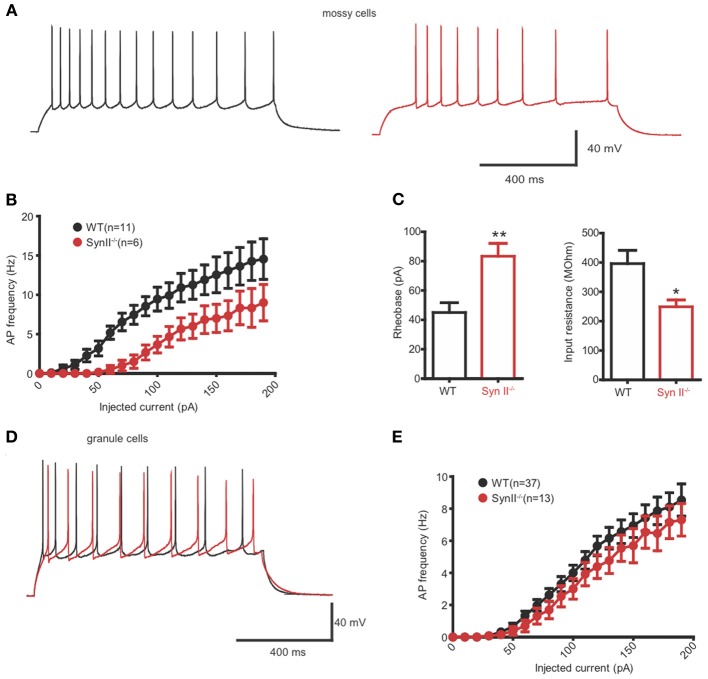
**Hilar mossy cells of pre-symptomatic Syn II^−/−^ mice display decreased excitability. (A)** Representative traces of whole cell current-clamp recordings from hilar mossy neurons in acute slices of WT (black) and pre-symptomatic Syn II^−/−^ (red) mice. **(B)** Action potential (AP) frequency plotted as a function of the injected current for both genotypes. **(C)** Mean (±s.e.m.) rheobase and input resistance; ^*^p < 0.05, ^**^p < 0.01, two-tailed unpaired Student's *t*-test. **(D,E)** Representative traces of whole cell current-clamp recordings from granule neurons **(D)** in acute slices of WT (black) and pre-symptomatic Syn II^−/−^ (red) mice and AP frequency **(E)** plotted as a function of the injected current for both genotypes.

Extracellular fPSPs are the summation of a series of events, notably synaptic activity and synchronous firing of APs by groups of neurons (Buzsaki et al., 2012). Thus, in the next experiment, we evaluated the firing rate of hilar mossy neurons from pre-symptomatic Syn II^−/−^ in the current clamp configuration. In the presence of specific antagonists that fully block synaptic activity, mossy cells were injected with 30 current steps, lasting 1 s and ranging from −100 to +200 pA, in 10 pA increments (Figures 5A,B). The firing rate of mossy neurons was lower in Syn II^−/−^ slices with respect to WT recordings (Figure 5B) and was accompanied by a significant increase in the rheobase (45.0 ± 6.7, *n* = 11 neurons/5 mice for WT vs. 83.3 ± 8.8 for Syn II^−/−^, *n* = 6 neurons/4 mice; two-tailed unpaired Student's *t*-test, *p* = 0.003) (Figure 5C, left). Input resistance, a parameter correlated with the firing rate, was also significantly reduced in Syn II^−/−^ (396.0 ± 45.2 MΩ, *n* = 11 neurons/5 mice for WT vs. 248.7 ± 23.2 for Syn II^−/−^, *n* = 6 neurons/4 mice; two-tailed unpaired Student's *t*-test, *p* = 0.031) (Figure 5C, right). On the contrary, recording from granule cells revealed no differences in the firing rates of WT and Syn II^−/−^ neurons (Figures 5D,E), with no genotype-dependent difference in either input resistance or rheobase (data not shown). Other intrinsic membrane properties (resting and threshold potential, AP amplitude, half-amplitude width, after-hyperpolarization current) were similar for the two genotypes in both mossy and granule neurons (data not shown). These results indicate that the reduced fPSPs in the hilar region of pre-symptomatic Syn II^−/−^ mice (Figure 1) are associated with reduced excitability of Syn II^−/−^ mossy cells, but not granule cells.

### Adult Syn II^−/−^ hilar mossy cells recapitulate the phenotype of pre-symptomatic Syn II^−/−^ mice

**Figure 6:**
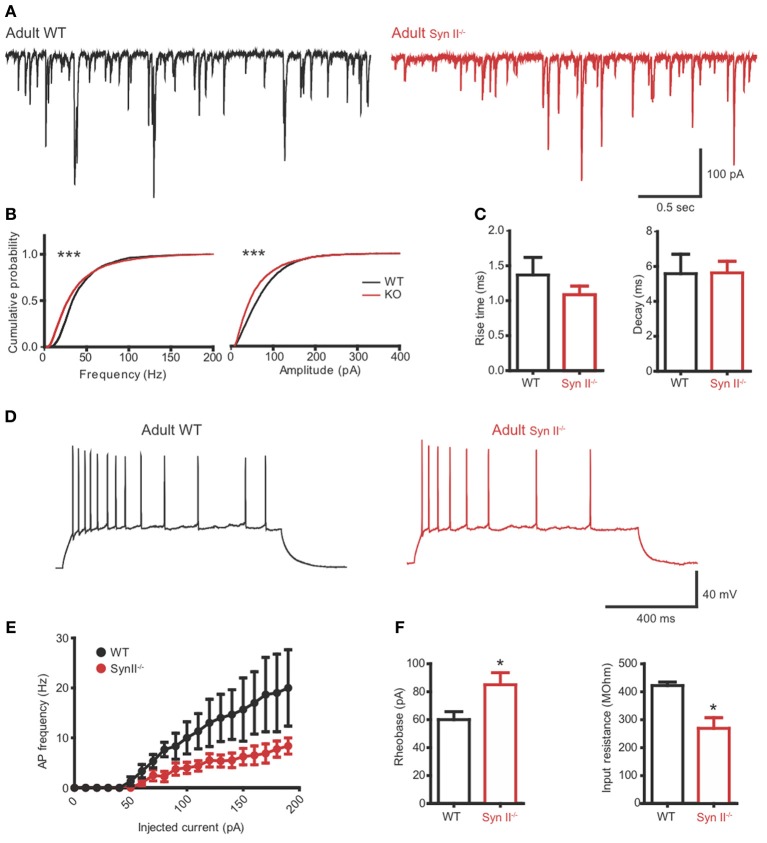
**The hilar mossy cell phenotype of pre-symptomatic Syn II^−/−^ mice is maintained in adult symptomatic Syn II^−/−^ mice. (A)** Representative mEPSC traces **(A)** and cumulative distributions **(B)** of their amplitude and frequency from 4 to 6 months old WT (black) and Syn II^−/−^ (red) mossy cells; ^***^p < 0.001, Kolmogorov–Smirnov test. **(C)** Mean (±s.e.m.) rise-time (10–90%) and mono-exponential τ of decay of mEPSCs from 4–6 months old WT (black bars) and Syn II^−/−^ (red bars) neurons. **(D)** Representative traces of current-clamp recordings from hilar mossy neurons in acute slices of WT (black) and symptomatic Syn II^−/−^ (red) mice. **(E)** Frequency of APs plotted as a function of the injected current for both genotypes. **(F)** Mean (±s.e.m.) rheobase and input resistance; ^*^p < 0.05, two-tailed unpaired Student's *t*-test.

To verify if the reduced excitability of hilar mossy cells in pre-symptomatic Syn II^−/−^ slices persists after the initiation of epileptic seizures in these mice, we repeated the experiments from Figures 4, 5 on adult (4–6 months old) Syn II^−/−^ mouse slices. As in pre-symptomatic mice, both the amplitude and the frequency distributions of mEPSCs were significantly shifted toward lower values in Syn II^−/−^ hilar mossy neurons (*n* = 4 neurons/3 mice for WT and 6 neurons/3 mice for Syn II^−/−^; Kolmogorov–Smirnov test, *p* < 0.001) (Figures 6A,B). The smaller amplitude distribution of Syn II^−/−^ cells was not accompanied by any change in the kinetic parameters of the response with respect to the WT (rise-time 10–90%: 1.36 ± 0.2 vs. 1.08 ± 0.1 ms, *p* = 0.296; decay *τ*: 5.58 ± 1.1 vs. 5.63 ± 0.6 ms, *p* = 0.967; two-tailed unpaired Student's *t*-test) (Figure 6C). Moreover, the firing rate of mossy cells was lower in adult Syn II^−/−^ (Figures 6D,E), with a significant increase in rheobase (60.0 ± 5.7, *n* = 3 neurons/3 mice for WT vs. 85.0 ± 8.6 pA for Syn II^−/−^, *n* = 8 neurons/3 mice; two-tailed unpaired Student's *t*-test, *p* = 0.043) (Figure 6F, left) and a decrease in input resistance (422.0 ± 12.5 MΩ, *n* = 3 neurons/3 mice for WT vs. 269.0 ± 38.0 for Syn II^−/−^, *n* = 8 neurons/3 mice; two-tailed unpaired Student's *t*-test, *p* = 0.042) (Figure 6F, right). These results show that the cellular phenotype of Syn II^−/−^ mice appears long before the appearance of an overt epileptic phenotype.

### The inhibitory output of hilar mossy cells to granule cells is reduced in both pre-symptomatic and symptomatic Syn II^−/−^ mice

**Figure 7:**
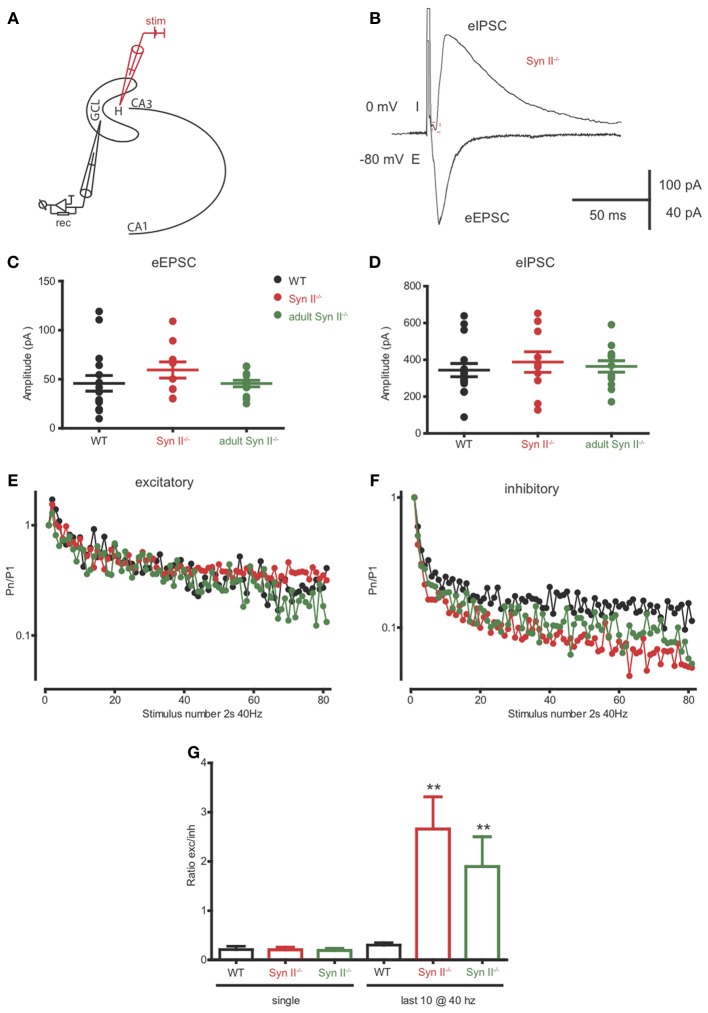
**The inhibitory output of hilar mossy cells to granule cells is reduced in both pre-symptomatic and symptomatic Syn II^−/−^ mice. (A)** Scheme of the experimental setup. **(B)** Representative traces of an eEPSC (−80 mV, inward) and an eIPSC (0 mV, outward) recorded in the voltage-clamp configuration from the same pre-symptomatic Syn II^−/−^ granule cell after the stimulation of the perforant path. **(C,D)** Aligned dot-plots representing the amplitude of the eEPSCs **(C)** or eIPSCs **(D)** from young WT (black), pre-symptomatic (red) and symptomatic (green) Syn II^−/−^ mice. **(E,F)** Plots of the normalized mean amplitude of excitatory **(E)** or inhibitory **(F)** responses vs. time showing the multiple-pulse depression during a 2-s train at 40 Hz in young WT (black), pre-symptomatic (red) and symptomatic (green) Syn II^−/−^ mice. **(G)** Mean (±s.e.m.) ratio between the amplitudes of eEPSCs and eIPSCs from the same granule neurons (young WT, black; pre-symptomatic Syn II^−/−^, red; symptomatic Syn II^−/−^, green) in the case of a single stimulus (left) or for the last 10 stimuli in a 40 Hz train (right); ^**^p < 0.001, Kruskal–Wallis test followed by Dunn's multiple comparison test.

The axons of hilar mossy cells project into the inner molecular layer of the DG, where they make excitatory synapses directly with granule cells, or with GABA interneurons, leading to disynaptic inhibition of granule cells (Scharfman and Myers, 2012; Jinde et al., 2013). Since Syn II is abundantly and specifically expressed in the mossy cell terminals of the inner molecular layer (Figure 3), the next step was to evaluate the net effect of the mossy cell output on granule cell activity. To this aim, we patched granule neurons from the granule cell layer and stimulated the axons of mossy cells in the region below the granule layer (Figure 7A). When granule neurons are voltage-clamped at -80 mV, close to the reversal potential of Cl^−^, the stimulation should result in an evoked excitatory inward response non-contaminated by inhibition. On the contrary, when the clamped voltage is shifted to 0 mV, the Cl^−^ drive will be predominant, and the stimulation should elicit a net inhibitory, outward response representing the fast-forward inhibition resulting from the intermediate activation of GABA interneurons (Figure 7B). Single stimulation did not reveal any difference between the amplitude of both eEPSC and eIPSCs in WT and pre-symptomatic and adult Syn II^−/−^ slices (*n* = 16 neurons/6 mice for WT, 10 neurons/4 mice for young Syn II^−/−^ and 13 neurons/3 mice for adult Syn II^−/−^; One-Way ANOVA followed by the Bonferroni's multiple comparison test, *p* = 0.344 and 0.751 for eEPSCs and eIPSC, respectively) (Figures 7C,D). Instead, the application of a 40 Hz tetanic stimulation revealed that depression was significantly increased at inhibitory synapses in both young and adult Syn II^−/−^ granule neurons (Figure 7F), while it was similar between genotypes at excitatory synapses (Figure 7E). We quantified this effect by measuring the ratio between the evoked excitatory and inhibitory responses (E/I ratio) on the same granule cell. Single pulse stimulation of the mossy cell axon produced postsynaptic inhibitory and excitatory currents whose ratio was similar between genotypes (*p* = 0.938; One-Way ANOVA followed by the Bonferroni's multiple comparison test) (Figure 7G, left). However, when the E/I ratio was measured for the last 10 responses in the train, it was significantly increased in both pre-symptomatic and symptomatic Syn II^−/−^ slices, with a decrease of inhibitory responses (E/I ratio = 0.3 ± 0.04 for WT, *n* = 6 neurons/3 mice; 2.6 ± 0.6 for young Syn II^−/−^, *n* = 7 neurons/4 mice; 1.8 ± 0.6 for adult Syn II^−/−^, *n* = 6 neurons/3 mice; *p* = 0.0011; Kruskal–Wallis test followed by the Dunn's multiple comparison test) (Figure 7G, right). This change in the ratio between excitation and inhibition may be responsible for the hyperexcitability of the DG under conditions of sustained high frequency synaptic input, as seen in Figure 2.

## Discussion

Epilepsy affects 1% of the general population, and 0.5% of children (Cowan, 2002). In the last 15 years, with the advent of genetic screening of epileptic families, many causative mutations in epileptogenic genes have been identified. The vast majority of these genes code for ion channels, or ion channel auxiliary subunits, with Na^+^ channels as the main “actors” (Gardiner, 1999; Poduri and Lowenstein, 2011). Some mutations have been also found in genes that encode proteins involved in the presynaptic SV release machinery or cell metabolism (Cavalleri et al., 2007; Striano et al., 2008; Suls et al., 2009; Pearl et al., 2011). As a result of these findings, more than 15 transgenic and knockout mouse models have been generated to aid in the study of epilepsy (Mantegazza et al., 2010). These models are superior to pharmacologically induced models. They often closely resemble the human pathology and offer the possibility to study the evolution of the disease and test new therapeutic strategies. These organisms can be studied in the pre-symptomatic phase, to investigate the mechanisms that lead to epileptogenesis, and distinguish them from subsequent secondary mechanisms that lead to the aggravation of symptoms.

As mentioned above, *SYN2* alterations in humans seem to confer predisposition for epilepsy. Previous studies have suggested an association of *SYN2* rs3773364 A>G polymorphism with febrile seizures in the UK, Irish, and Finnish cohorts (EPIGEN Epilepsy Genetic Consortium; Cavalleri et al., 2007) and in Indian patients with idiopathic epilepsy (Lakhan et al., 2010), but not in the Australian cohort (Cavalleri et al., 2007) or in Malaysian epileptic patients (Haerian et al., 2011). Remarkably, mice lacking one or more Syn isoforms are all prone to epileptic seizures, with the exception of Syn III^−/−^ mice (Rosahl et al., 1995; Cesca et al., 2010; Etholm et al., 2011, 2012; Ketzef et al., 2011; Farisello et al., 2013). The model used in the present study is the Syn II^−/−^ mouse developing an overt epileptic phenotype around the age of 2 months (Bogen et al., 2011; Etholm et al., 2011, 2012). It remains unclear why absence of Syn I or II leads to hyperexcitability. An early study (Rosahl et al., 1995) investigated mice lacking Syn I, Syn II, or both isoforms. By using extracellular recordings from CA1 pyramidal neurons, it was shown that paired-pulse facilitation was increased in the Syn I^−/−^, but not in the Syn II^−/−^ or in the double Syn I/II^−/−^ mouse. On the contrary, post-tetanic potentiation was decreased in the latter two genotypes, but not in the Syn I^−/−^ mouse. The Syn II^−/−^ and Syn I/II^−/−^ mice also underwent severe synaptic depression upon repetitive stimulation (Rosahl et al., 1995).

We have recently shown that pre-symptomatic Syn I/II/III^−/−^ mice display an impaired tonic current, due to defects in GABA release and spillover and leading to diffuse hyperexcitability of hippocampal pyramidal neurons (Farisello et al., 2013). A similar study (Ketzef et al., 2011) showed that, increased field responses were elicited in these mice during the pre-symptomatic phase. They further suggest that in pre-symptomatic animals a number of compensatory mechanisms take place that enhance both inhibition and excitation, and eventually culminate with the onset of seizures. In a parallel study (Boido et al., 2010), the action of the antiepileptic drug levetiracetam was investigated in Syn I/II/III^−/−^ mouse slices. They used 4-aminopyridine to evoke epileptic-like events and showed that levetiracetam ameliorated abnormal activity more efficiently in WT than in Syn I/II/III^−/−^ mice. This finding can be explained by the decreased levels of the levetiracetam receptor SV2A due to the marked decrease in SV density observed in these animals (Gitler et al., 2004).

The basic question is why this phenotype appears so late in post-natal development, if other phenotypic traits, such as the sharp loss of the reserve pool of SVs in nerve terminals (Baldelli et al., 2007; Cesca et al., 2010; Lignani et al., 2013), are altered much earlier. This view has been extended by more recent studies showing that Syns are actively involved in post-docking steps of exocytosis, delivering SVs to the active zones and affecting the kinetics and synchronization of release (Hilfiker et al., 1998; Bykhovskaia, 2011; Medrihan et al., 2013). In the case of Syn II, it is interesting that the protein expression profile increases from birth to reach a plateau around postnatal day 60, coinciding with the onset of seizures (Bogen et al., 2011). Thus, it is possible that the overt epileptic phenotype appears only when the synapses require the full expression of Syns to maintain a proper balance between excitatory and inhibitory transmission. Moreover, epileptogenesis is a multifactorial disorder that involves a cascade of molecular, cellular and network alterations occurring over a long time interval after the initial insult (Rakhade and Jensen, 2009; Hunt et al., 2013). The latent phase of epileptogenesis lasts months to years in humans, and between 2 and 12 weeks in rodent models (Rakhade and Jensen, 2009). In the case of a genetic model as Syn II^−/−^ mice, the synaptic dysfunctions during the first 2 months of life hinder the development of a proper balance between excitation and inhibition, eventually leading to seizures. Another important developmental aspect is the expression of GABA receptors and GABA synthetic enzymes that do not reach full expression until before the fourth week of life in rats. Furthermore, it is well known that in the first weeks of life GABA acts as an excitatory neurotransmitter, due to the increased intracellular Cl^−^ concentration (Rakhade and Jensen, 2009). These aspects could explain the late seizure onset in our mouse model. Although mossy cells have a decreased output and a lower excitatory drive on inhibitory interneurons, this does not significantly alter the interneuron output, since GABA-mediated inhibition is initially weak. This is probably a simplistic explanation, since a number of ion channels, ion pumps and neurotransmitter synthases reach full expression a few weeks after birth in rodents, making it a difficult task to pinpoint one single event that is crucial in seizure onset.

A specific characteristic of temporal lobe epilepsy is the loss of hilar neurons in the hippocampus. It was assumed for a long time that mossy cells are the main population to decrease in number, despite the fact that, until very recently, no specific marker to distinguish them from inhibitory interneurons was available. Recent results have shown that hilar mossy cells and GABA-positive interneurons are equally decreased in number in a head trauma epilepsy model (Santhakumar et al., 2000), suggesting that mossy cell survival might be as important as their loss in increasing seizure propensity. Currently, there are several theories regarding the reason why mossy cell loss could lead to hippocampal hyperexcitability. The first one is the “*cell loss-induced axonal sprouting*” hypothesis (Wenzel et al., 2000). It is based on the fact that granule cell axons massively innervate mossy cells. A partial loss of mossy cells, by triggering mossy fiber sprouting, would abnormally innervate other granule cells and thus create recurrent excitation loops. The second theory is the “*dormant basket cell*” hypothesis (Sloviter, 1991). This hypothesis proposes a role for mossy cells in exciting the inhibitory basket cells, present in the granule cell layer. A decrease in the number of mossy cells would then lead to a hypoactivation of inhibitory interneurons and to dentate granule cells hyperexcitability. The third theory, the “*irritable mossy cell*” hypothesis, emphasizes the role of the surviving mossy cells that would undergo various alterations, leading to the amplified activity of the granule cells (Scharfman and Myers, 2012). A more recent theory integrates the latter two (Scharfman and Myers, 2012), basing itself on anatomical data and paired recordings showing that mossy cells synapses preferentially target interneurons locally (Scharfman, 1995) and have projections that extend along the septo-temporal hippocampal axis which mainly synapse on granule cells (Buckmaster et al., 1996). Furthermore, it seems that mossy cells do not connect only with basket cells, but with a range of different inhibitory interneurons also in the hilus. Thus, the role of mossy cells in regulating hippocampal activity is rather complex and difficult to predict.

It is not known which, if any, of these theories represents the true mechanism by which the hippocampus becomes hyperexcitable. An essential aspect is the fact that mossy cells convey feedback from the CA3 pyramidal neurons to the granule cells. This circuit configuration might provide the frame for reverberant activity and the transition from brief, interictal events, to seizure-like activity (Scharfman et al., 2001). To test any of the above-described theories, one would need a tool to specifically eliminate mossy cells from the hippocampus and study the subsequent changes. This experiment was performed by Jinde et al. (2012), who generated a conditional transgenic mouse selectively expressing the diphtheria toxin receptor in the mossy cells. After a few weeks of diphtheria toxin injection in the DG, massive mossy cell degeneration was observed, accompanied by a transient increase in theta power during exploration, deficits in contextual discrimination, and increased anxiety. Patch-clamp recordings of granule cells showed that the frequency, but not the amplitude, of both sEPSCs and sIPSCs was decreased (Jinde et al., 2012). These results strengthen the view that mossy cells convey both direct excitatory and indirect/feed-forward inhibitory input to granule cells, and that the balance between these two inputs plays a key role for the spatial and temporal control of granule cell excitability in the DG. Nonetheless, the animals did not exhibit any epileptiform activity (Jinde et al., 2012), confirming that, as it is in our case, the hypoexcitability of mossy cells is not the sole culprit for epileptic hippocampal activity.

In the present study, we dissected the local expression of Syn II in the mouse hippocampus and showed that it is enriched in regions were granule cell axons project, as well as in the inner molecular layer, where inhibitory synapses from the hilus and granule cell interneurons, and excitatory projections from hilar mossy cells are found. Notably, in this area, the, expression of the mossy cell marker calretinin overlapped with the expression of Syn II (Figure 3). Upon single stimulation of the perforant path in pre-symptomatic Syn II^−/−^ mice we found a decreased field response and a longer latency in the hilus, suggesting impairments in the synaptic release mechanism at mossy fiber terminals and/or a decreased responsiveness of hilar cells. Moreover, synaptic depression in response to tetanic stimulation was significantly decreased in the granule cell layer of the Syn II^−/−^ mice. Based on previous work showing that perforant path activation is similar in Syn II^−/−^ and WT age matched mice (Medrihan et al., 2013) and considering that somatic granule cell properties were unchanged, we suggest that these changes arise from an altered feedback received from the hilar region. Indeed, patch-clamp recordings of mossy cells demonstrated that the dysfunction lies on both sides: on one hand, vesicular release from mossy fibers is reduced, and on the other hand, mossy cell excitability is reduced. These changes appeared before the overt epileptic phenotype and persisted in the adulthood. Finally, the dissection of the excitatory and inhibitory inputs to granule cells from mossy cells revealed that, under conditions of sustained high frequency synaptic activity, the excitation/inhibition ratio is significantly increased in both pre-symptomatic and symptomatic Syn II^−/−^ mice.

It is difficult to explain why the loss of a presynaptic protein leads to decreased excitability of mossy cells. The latter could be the expression of a homeostatic mechanism operating at the level of the hippocampal network that fights against hyperexcitability and absolutely requires the full expression of Syn II when plasticity in the adult brain decreases. This hypothesis suggests that one of the functional roles of Syns I and II, whose expression strongly increases after the first month of postnatal life and remains high throughout adulthood (Lohmann et al., 1978; Bogen et al., 2009), is to provide a high degree of plasticity to the adult synapses.

The functional changes in the DG of the Syn II^−/−^ may not be all due to the sole absence of synapsin and high-throughput profiling studies will prove useful to investigate the relative expression of other genes/proteins involved in epileptogenesis. However, these studies would be difficult to design in our case, since the absence of synapsin II may have developmental long term effects on many aspects of the maturation of the hippocampal network. The profiling studies are usually investigating the changes induced by the application of a drug or trauma or a mutation in a transcription/repressor factor that can directly lead to changes in the transcription machinery (Bando et al., 2011; Pernot et al., 2011; Goldberg and Coulter, 2013). In our case any change in DNA/RNA/proteins might reflect adaptive responses to the primary changes in synaptic transmission and network excitability induced by the synapsin II mutation. Indeed, the Syn II^−/−^ mouse is characterized, like all other synapsin mutants, by a severe reduction in the density of the SVs, leading to a general decrease in the expression of SV proteins, such as synaptophysin I, SV2 and Rab5 (Rosahl et al., 1995).

Overall, our results reinforce the idea that mossy cells have a predominantly inhibitory effect within the hippocampus, through feedback inhibition on granule cells and that impairments in their function could trigger, or participate in an excitation/inhibition imbalance at the level of the DG of the hippocampus that represents a key factor for epilepsy predisposition. However, epileptogenesis is a multifactorial process that follows a precise temporal sequence of events in most animal models of epilepsy (pilocarpine, kindling, trauma) leading to progressive neural circuitry alterations (Rakhade and Jensen, 2009; Hunt et al., 2013). Thus, our findings suggest that the hypoexcitability of mossy cells is just one factor among others that weakens the DG circuitry and renders it more prone to hyperexcitability in the animal adult. Studies on experimental models of epilepsy, such as the Syn II^−/−^ mouse, could shed light on the complex synaptic mechanisms orchestrating network excitability and contribute to ameliorate diagnosis and prognosis of idiopathic epilepsy linked to synaptic abnormalities.

### Conflict of interest statement

The authors declare that the research was conducted in the absence of any commercial or financial relationships that could be construed as a potential conflict of interest.
